# Clinical research for life-threatening illnesses requiring emergency hospitalisation: a critical interpretive synthesis of qualitative data related to the experience of participants and their caregivers

**DOI:** 10.1186/s13063-023-07183-6

**Published:** 2023-02-28

**Authors:** David S. Lawrence, Agnes Ssali, Joseph N. Jarvis, Janet Seeley

**Affiliations:** 1 Department of Clinical Research, Faculty of Infectious and Tropical Diseases, London School of Hygiene and Tropical Medicine, Keppel Street, London, UK; 2 Botswana Harvard AIDS Institute Partnership, Gaborone, Botswana; 3 Social Aspects of Health Across the Life-Course Programme, MRC/UVRI & LSHTM Uganda Research Unit, Entebbe, Uganda; 4 Department of Global Health and Development, Faculty of Public Health and Policy, London School of Hygiene and Tropical Medicine, London, UK

**Keywords:** Informed consent, Emergency, Clinical trial, Clinical research, Decision-making, Review, Qualitative research

## Abstract

**Background:**

Research into life-threatening illnesses which require emergency hospitalisation is essential. This group of patients is unique in that they are experiencing an unfolding emergency when they are approached, enrolled, and followed up in a research study. We aimed to synthesise qualitative data from trial participants and surrogate decision-makers to deepen our understanding and inform the design and conduct of future clinical trials for life-threatening illnesses.

**Methods:**

We conducted a critical interpretive synthesis of qualitative data from trial participants and surrogate decision-makers related to the experience of participating in a clinical research study when suffering from a life-threatening illness. A scoping review informed a systematic review of published data. We searched research databases and reviewed papers for inclusion. Primary data and interpretations of data were extracted from each paper. Data were analysed using reciprocal translational analysis, refutational synthesis, and lines of argument synthesis to develop a synthetic construct.

**Results:**

Twenty-two papers were included. Most individuals had no previous knowledge or experience with clinical research. Individuals making decisions were directly experiencing or witness to an unfolding emergency which came with a myriad of physical and psychological symptoms. It was difficult to differentiate clinical research and routine care, and understanding of core concepts around research, particularly randomisation and equipoise, was limited. We found that this led to an underestimation of risk, an overestimation of benefit, and an expectation of being allocated to the intervention arm. The decision-making process was heavily influenced by trust in the research team. Individuals suggested that abbreviated information, presented in different ways and continuously throughout the research process, would have increased knowledge and satisfaction with the research process.

**Conclusion:**

Individuals suffering from a life-threatening illness who are being invited to participate in clinical research need to be managed in a way that adapts to the severity of their illness and there is a need to tailor research processes, including informed consent, accordingly. We provide suggestions for further research and implementation work around research participation for individuals suffering from a life-threatening illness.

**Trial registration:**

PROSPERO CRD42020207296

**Supplementary Information:**

The online version contains supplementary material available at 10.1186/s13063-023-07183-6.

## Background

Clinical trials are essential to determine how to manage illness and improve lives. Randomised controlled trials are recognised as the gold standard in the generation of medical evidence and are a primary source of data when generating treatment guidelines. Interventional clinical trials would not be possible without the willing participation of individuals who are suffering with the illness under investigation. Conventionally, all prospective participants for a clinical trial must be fully informed of the study and be willing to provide consent, free from coercion; however, in some scenarios, research does take place with either deferred consent or through waivers of consent. Once enrolled, participants move through a series of processes which may include the provision of personal and medical information, physical examination, investigations such as blood tests or imaging, administration of an intervention such as medication, and ongoing follow-up to measure or determine their response. All participants are free to withdraw their consent at any time during the course of the study and can do so without having to provide a reason. These processes are guided by ethical principles laid out by the Declaration of Helsinki [1] and the International Conference on Harmonisation Good Clinical Practice [2].

Qualitative methods research is often conducted alongside clinical trials, both to measure the personal, psychological, or ‘quality-of-life’ outcomes of an intervention but also more broadly to explore bioethical aspects of clinical research. This work has focused particularly on the motivation for participating in trials, experience of the informed consent process, and participant satisfaction with the trial experience as a whole.

### Motivation

There has been much research conducted as to the underlying motivation for joining clinical trials [3–8]. Clinical trials are primarily designed to answer a research question, the findings of which it is hoped will later be of benefit to a larger population. The concept of ‘therapeutic misconception’ is well documented in clinical research and is the belief that every aspect of the research project to which someone has consented has been designed to benefit them directly [9]. Some individuals may benefit by participating but this research is not designed so that everyone will [10]. Despite this, it is not uncommon for research participants to expect a personal therapeutic benefit from the treatment they receive, including in placebo-controlled trials [11, 12]. Altruism is also a factor but may be described as being ‘conditional’ on receiving these personal benefits [6–8].

### Informed consent

The process of informed consent has been subject to much scrutiny by clinical trialists and social scientists alike. Current approaches to consent frame patients as active decision-makers and can exaggerate their agency [13]. ‘Doing consent’ is seen as an easily auditable process which protects researchers rather than participants [4], and as a result, discussions around the ethics of informed consent often focus on information provision and the readability of forms [14]. Comprehension of the informed consent process, although not universally defined, has been well studied and found to be generally poor [15, 16], particularly where participant information sheets are considered too long and technical [4, 17–20].

### Participant experience

Understanding the participant experience as they navigate through the scheduled events of a clinical trial can provide an opportunity to improve ongoing trials and develop better trials for the future. A broad range of qualitative methods has been used to explore participant experience, ranging from interviews focused on ‘participant satisfaction’ [21] or ‘good participatory practice’ [22] to in-depth ethnographic studies adopting a range of theoretical perspectives [23, 24].

This review of qualitative methods research aims to explore participation in a clinical trial when an individual was suffering specifically from a life-threatening illness. We aim to synthesise the experience of participants and their loved ones who are recruited whilst suffering from a condition that has led them to be admitted to hospital and for which there is a risk of death. We believe that the severity of their underlying condition and the urgency with which treatment (and therefore enrolment) must be initiated create a complex sociological context. This context could have a unique impact on their motivation to participate, the informed consent process, and their perspective on the clinical trial experience as a whole. Given the high stakes of such a scenario, there is value in collating and synthesising qualitative data to understand how individuals navigate this process, make decisions, and reflect on the experience from beginning to end. This stands to deepen our understanding and inform the design and conduct of future clinical trials for life-threatening illnesses.

We therefore conducted a critical interpretive synthesis with the aim of collating data from the perspective of participants and their caregivers related to the experience of being in a clinical trial for a life-threatening illness.

## Methods

We conducted a critical interpretive synthesis broadly in line with the methodology outlined by Dixon-Woods et al. [25]. We acknowledged that there was significant heterogeneity in the methodology of published critical interpretive syntheses and that this approach has evolved over time [26]. We therefore adopted an approach to the methodology that was flexible and evolved to enable us to best meet our aim.

### Defining the population

We defined our population of interest as any individual (or their caregiver), regardless of age, diagnosed with a life-threatening illness and recruited into a clinical study. A life-threatening illness was defined as any medical condition that required emergency inpatient admission to a healthcare facility and for which the potential sequelae included death. A clinical study was defined as any prospective observational or interventional study that required the individual or a surrogate to provide consent. We wanted to begin to understand the entire experience from beginning to end so included studies exploring all aspects of the clinical study including being approached, screened, consented, randomised, managed, and followed up as a participant. Despite the differences compared to interventional studies, we opted to include observational studies as participants still need to move through most of these processes. We did however exclude clinical studies with a waiver of consent as despite not wanting to focus entirely on the consent process itself we were interested in experiences whereby individuals had been involved in a decision-making process. A systematic review of research without prior consent in paediatric trials has been published elsewhere [27]. We were solely interested in in-depth qualitative research published in English that related to the trial experience rather than that focused specifically on the acceptability of the intervention under investigation.

### Scoping review

An initial scoping review was conducted to identify published work that was relevant to the research question. Following Eakin and Mykhalosvsky [28], we reviewed and discussed a selection of relevant papers and then used this broad review as a basis to refine our comprehensive search strategy. We approached the concept of life-threatening illnesses by searching for broad terms such as ‘emergency’, ‘mortality’, and ‘life-threatening’ as well as a select number of pathologies that are deemed to be life-threatening such as ‘meningitis’ and ‘stroke’. During this process, we acknowledged that a broad range of pathologies and scenarios could technically be life-threatening and therefore accepted that any comprehensive search strategy was likely to produce a large number of results. From this initial scoping review, we were then able to define a comprehensive search strategy. The inclusion and exclusion criteria for the critical interpretive synthesis are presented in Table 1.

**Table 1:** Inclusion and exclusion criteria

Inclusion	Exclusion
Enrolled in a prospective (observational or interventional) clinical study that required the provision of consent	Involved in a retrospective study or did not need to provide consent
Clinical study focuses on a life-threatening condition	Not a life-threatening condition
Data from study participant or their caregiver/relative/surrogate/parent/guardian	Data from anyone else
Qualitative or mixed-methods study	Exclusively quantitative analysis
Semi-structured or in-depth interview, focus group, ethnography, observation, diaries	Self-administered, short answer or structured questionnaire, multiple-choice answer survey
Data relating to the trial experience	Data focusing on the intervention, data for secondary outcomes, e.g. acceptability
Full-length, original research paper	Abstracts, poster, conference proceeding, viewpoint, commentary
English	Not in English

### Comprehensive search

We developed a search strategy (Table S1) and searched the following information sources: MEDLINE, Embase, Web of Science, Global Health, JSTOR, Academic Search Complete, Scopus, African Journals Online, PsychINFO, and PsychEXTRA. There was no restriction on the publication date. Reference lists of included studies were also searched to identify any additional potentially eligible studies. All papers were then entered into Covidence and duplicates were removed. The titles and abstracts of all potentially eligible studies were screened by both DSL and AS to determine which were suitable for full-text review. DSL and JNJ are clinicians with specialist training in internal medicine and were able to provide a professional opinion on the life-threatening nature of the illness under study. In the case of disagreement, the two reviewers discussed and, if necessary, JS and JNJ were also available for arbitration. DSL and AS then reviewed the full text of those studies and the same arbitration approach was adopted to determine which would be included in the full review. When planning this stage, there was uncertainty around the number of papers that would be identified by the search and how many would be eligible for inclusion in the review. If faced with an unmanageable workload, we therefore considered drawing on purposive sampling and employing theoretical sampling and theoretical saturation to decide on a collection of papers that would be appropriate; however, this was not necessary.

### Data extraction and analysis

We developed a data extraction form (Table S2) with domains related to the focus of the clinical study, the methodology of the qualitative study, the results including any themes and their description, theoretical frameworks, and all primary data presented and a quality assessment. We extracted both primary data such as direct quotes as well as interpretive data including themes, frameworks, and conclusions. Where data were collected from a range of informants, we focused on the perspective from study participants and surrogate decision-makers, rather than researchers or those who declined to participate. We did not include those who declined as we were interested in the entire continuum of a clinical trial and that can only be elicited from those who have participated. DSL and AS extracted data from half of the included papers each, with the other then reviewing the data extraction form and amending after discussion, as necessary.

### Critical interpretive synthesis

Throughout the searching and extraction process, DSL and AS became increasingly familiar with the papers and the extracted data to develop a codebook. DSL coded the extracted data in NVivo 12 and AS did so manually. Together, they then met regularly and adopted three major strategies of meta-ethnography to support the interpretive synthesis: reciprocal translational analysis to identify the key themes or concepts in each paper as reported, refutational synthesis to identify any contradictions between study reports and attempt where possible to explain them, and lines of argument synthesis to build on interpretations that were found in the papers. This process then facilitated the development of a synthetic construct which aimed to broadly encompass the entirety of the critical interpretive synthesis.

As this was a review using published data, there was no requirement for ethical approval. The review was prospectively registered on PROSPERO (CRD42020207296).

## Results

**Fig. 1:**
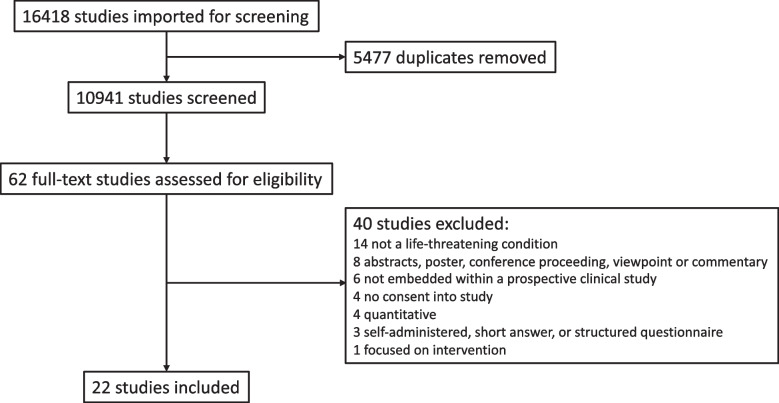
PRISMA diagram

The comprehensive search strategy took place on 12 and 13 November 2020 and the results of the process are presented in the PRISMA diagram (Fig. 1). 16,418 studies were imported for screening, and after removing duplicates, 10,941 underwent title and abstract review. A total of 62 papers underwent full-text review and 22 were included. No additional papers were included after reviewing the bibliographies.

### Summary of the papers

We identified 22 papers published between 1997 and 2019 (Table S3) [29–50]. Nineteen were conducted in high-income countries (eight in the UK [36–38, 42–44, 47, 48], four in the USA [33, 34, 41, 50], three in Canada [30, 31, 45], and one in each of Denmark [35], Norway [39], Sweden [29], and Switzerland [49]) and three in lower and middle-income countries (two in Ghana [32, 46] and one multi-site in Kenya and Uganda [40]). The qualitative methods studies were embedded within 18 RCTs [29, 30, 32–44, 47–49] and one within an observational study [46], with three embedded within intensive care units hosting a variety of different interventional and observational studies but not within a specific named study [31, 45, 50]. The populations of the parent study were adults in 14 studies [29–31, 33–39, 41, 47–49] and children and/or neonates in eight studies [32, 40, 42–46, 50]. The diseases studied included myocardial infarction and acute coronary syndrome [29, 33, 35, 41, 48], stroke [39, 41], chronic obstructive pulmonary disease [49], malaria [32, 46], severe febrile illness [40], post-partum haemorrhage [36], retained placenta [37, 38], and open fractures [47]. In studies where there was no focus on a specific pathology, the participants were all individuals admitted to intensive care units and were therefore undoubtedly suffering with a life-threatening illness [30, 31, 34, 42–45, 50].

Qualitative data were collected from a total of 668 participants. The informants within the qualitative methods studies were adult participants in 11 studies [29, 33, 35–39, 41, 47–49] and surrogate decision-makers—mainly parents—in 10 studies [30–32, 40, 42–46, 50], with one study interviewing both [34]. Where stated, the data collection for the qualitative methods studies took place from within a few days up to 18 months from enrolment into the parent study. Most papers used interviews for data collection which were subject to either thematic or content analysis. There were no major methodological weaknesses identified which precluded any of the papers from being included in this synthesis.

### The synthetic construct

**Fig. 2:**
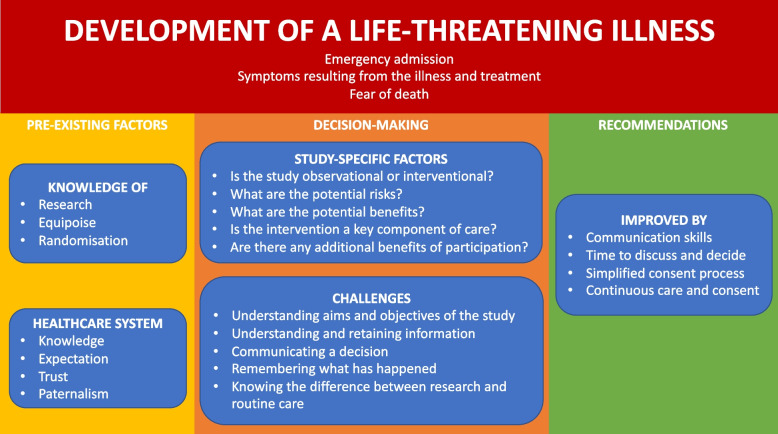
Synthetic construct

Our synthetic construct is presented in Fig. 2, and we will explain this in a relatively chronological format throughout the time course of a research study. Within this analysis, we will focus on five key domains. The first is the experience of suffering with a life-threatening illness which is overarching and permeates the subsequent four: pre-existing knowledge of research and expectations of healthcare, study-specific factors, challenges in the decision-making process, and recommendations for improvement.

### The experience of suffering with a life-threatening illness

Conducting clinical research within an emergency situation is the focus of this critical interpretive synthesis. Our aim was to try and understand the experience of participants and caregivers living through those moments and then apply this as a lens through which we could try and understand its impact on all aspects of research participation. As described, study participants were suffering from severe illnesses that could, and in some cases did, lead to death. In some situations, this would be an exacerbation of a previously diagnosed condition, but in many, it was an acute event which was completely unexpected and diagnosed for the first time or which occurred as a complication of a normal process such as childbirth. Participants shared their experience of often being rushed to a healthcare facility and thrown into a completely unfamiliar environment whilst suffering with acute symptoms of their illness. This may have been acute pain from a myocardial infarction [29, 33, 35, 48] or a road traffic accident [47], breathlessness from a respiratory illness [49], septic shock from an overwhelming infection, or severe bleeding due to a post-partum haemorrhage [36] or retained placenta [37, 38]. These are symptoms which are uncomfortable and distressing and which can cause difficulty in understanding and retaining information as well as impairing communication such as asking questions and communicating decisions. This impairment may be due to distraction caused by fear [33, 43, 48, 50] or abnormal mental function as a result of the underlying pathology. In addition, individuals rapidly undergo invasive procedures such as the insertion of intravenous lines and are initiated on emergency treatments which aim to alleviate their symptoms and manage their diagnosis but which can cause discomfort and disorientation such as strong analgesia for severe pain [35, 47]. All of this process takes place within an accelerated period of time whereby diagnosis and initiation of treatment need to take place rapidly in order to improve the chance of survival, which itself is not certain. When considering this from the perspective of a surrogate decision-maker, they are witness to these events, and in the case of neonatal research, the decision-makers may have also been through a traumatic childbirth experience from which they are still recovering [42, 44].

Having framed the acuity of the situation and the rapid emergence of a life-threatening diagnosis with its accompanying symptoms and potential treatment-related side-effects, we now consider how this can impact on the experience of being in a clinical study.

### Pre-existing knowledge of research and expectations of healthcare

Before the development of a life-threatening illness and being approached to enrol in a clinical study, individuals already have their own pre-existing knowledge of research. We view these factors as laying the foundation upon which an individual makes a decision to enrol. We found that there were generally very low levels of awareness and understanding of the principles of clinical research prior to being approached to enrol and the vast majority of individuals did not have any previous first-hand experience of clinical research. This means that core principles such as equipoise and randomisation as well as broader issues such as how clinical trials are organised and implemented alongside routine care were poorly understood. These factors are independent of the life-threatening nature of the illness as they precede it. Few individuals had previous experience of research; however, we found prior research experience to be more common in resource-limited settings where parents had often enrolled multiple children in several research studies. Those who did have previous experience framed this as a positive reason to contribute [34, 45, 46].

Individuals also present to healthcare facilities with their own pre-existing experience of and relationship with healthcare. Some may present with exacerbations of chronic conditions that are already managed within primary care, sometimes with previous episodes of hospitalisation, whereas others may suffer from an initial presentation of a life-threatening illness which is being diagnosed for the first time. Expectations of different healthcare facilities and professionals may come directly from first-hand experience as a patient or a caregiver or indirectly via second-hand information from friends and family, or more broadly through exposure to external sources such as the government or the media. These expectations are crucial when it comes to determining how much trust to place in both the routine care and the research environment. For example, where an individual has low expectations of the routine care provided and is aware that research groups have access to greater resources, then this may lead them towards agreeing to participate in a research study. This was observed particularly in research studies conducted in resource-limited settings [32, 40]. However, in all settings, it is often difficult to disentangle routine care from research and therefore it becomes more difficult to understand the potential added benefits of being part of a research study, if they exist. Conversely, suspicions about research as a form of experimentation by using people as a ‘guinea pig’ [29] or as a means to obtain blood samples for illicit testing reduced trust [32].

The expectation of healthcare professionals specifically, whether based on prior experience or not, was found to be crucial in both the decision-making process and the broader experience of the research. Trust was a core concept that permeated throughout. When faced with a life-threatening illness, individuals explained that although it was not always possible to understand and digest the information, they often defaulted to agreeing to participate based on trust in the research team approaching them [29]. Where there was awareness of broader research infrastructure, there were also expressions of trust in research ethics committees and research institutions which were felt to provide safeguards through their regulatory procedures [39, 45]. Some individuals explained that they thought the researchers were the experts and knew best and that it seemed pointless to be asked their opinion with regards to enrolment as they knew so little about the subject themselves [39, 40]. We therefore found that in an emergency scenario, trust in healthcare workers was of paramount importance and influence. In contrast, we observed that in some settings where the healthcare system is more paternalistic, there would be a similarly passive approach towards decision-making which we found to be based more on acquiescence than coercion.

### Study-specific factors

Despite the above, we found that the decision-making process was highly impacted by several factors related to the research study specifically. The first is whether the study was observational or interventional. There was a small number of observational studies included within this synthesis and those with combined interventional and observational studies did not disaggregate their findings by study design. However, within the single observational study and our interpretation of the conclusions of papers with combined study designs, we identified that there were fewer concerns about the risks of participation simply because these only involved collection of data and/or specimens. We found that in the context of a life-threatening illness this was both seen as a positive because of the reduced risks and as a negative because of the potential inconvenience or discomfort of participating when an individual expects no personal, health-related gain through participation. It was when considering these observational studies that we were able to understand more how individuals felt about providing blood samples as these were often the primary focus of the research. Here we found that it was important to explain the purpose of taking blood samples, what they would be tested for, and why there may not be any immediate results available [46]. In terms of avoiding unnecessary discomfort, additional blood samples taken when venepuncture was being conducted for another reason were deemed more acceptable than taking a specific blood sample just for research purposes [45].

When considering interventional studies, we found that discussions around risk and benefits were more prevalent given the potential for the study to impact directly on the life-threatening illness. Given that the worst possible outcome of the illness was death, it was important to understand how the treatment being offered could improve the chances of survival. The potential benefits of the study were often felt to be immense, and in many instances, we found that decision-makers expected there to be a direct effect on them or the person they were representing [31] and the decision to enrol was made without hesitation [43]. This was true even in scenarios whereby the intervention itself was not necessarily expected to improve survival [34]. It was also true in trials of an intervention versus a standard of care where it was naturally expected that half of all participants would receive no additional benefit at all due to a lack of awareness of the concept of randomisation [42]. When considering risk, we found that the overriding trust in the research team and the wider research infrastructure meant that there was little consideration given to the possibility that the intervention could actually cause harm, rather that it might make no difference at all [44, 50]. As a result, we conclude that the focus was more towards the potential benefits than the risks.

When considering risks and whether to participate, we found that the nature of the intervention being studied was also of great importance. Where the intervention was perceived to be clearly related to the underlying pathology and was directly addressing the main problem, such as a blocked coronary artery, then the potential benefits were amplified [33]. This was still the case but to a lesser extent when considering if the intervention could avert something felt to be important but was not lifesaving, such as avoiding having surgery or reducing the length of a hospital admission [37, 48]. However, when the intervention was perceived to be of less importance to the bigger picture, such as the type of dressing applied after a major operation to repair an open fracture, then the potential benefits and risks were deemed to be smaller and the gravity of the decision was reduced [47, 50]. When risks were perceived, rightly or wrongly, to be low or absent, then it was articulated as there being nothing to lose and potentially something to gain if the intervention proved to be efficacious.

In addition to the impact of the intervention on health, there was also consideration given to any additional benefits of participation. These may be health-related, such as optimised management concomitant diagnoses, or financial in terms of transport reimbursement and financial incentives [47]. We found these to be more prevalent in research conducted in resource-limited settings but they were not interpreted as being prevailing factors in the decision-making process which was driven much more by a desire to survive [32].

Given the above, in the context of a life-threatening illness, we found that in general individuals expressed a strong desire to participate for a personal health benefit rather than from any more altruistic motive such as generating important scientific information or benefiting future patients because of the urgent, personal situation they faced. Where the risks and benefits were felt to be minimal, the decision was sometimes articulated as being made more in ambivalence or due to altruistic motives [48, 49].

### Challenges in the decision-making process

As well as considering the study-specific factors, there were additional aspects of the decision-making process that were exacerbated by having a life-threatening illness. The first of these was that it was harder to understand the aims, objectives, and procedures of the research. This was articulated directly in some cases but also interpreted to be the case in others. In the most extreme scenarios, participants reflected that they did not consider themselves competent to understand the information or to be able to make an autonomous decision in that particular situation saying that they ‘signed without understanding anything’ [29] and/or that they had forgotten about the study entirely [33, 35, 36]. In others, participants had not understood that enrolment was voluntary [39].

As discussed earlier, there was limited pre-existing knowledge about how clinical research works and therefore limited foundations from which to build when inviting individuals to participate. However, the severity of the unfolding situation made it harder for individuals to receive, retain, and weigh up information in the limited time they had to do so. This was particularly important when considering two factors: equipoise and randomisation. All interventional trials must have equipoise, an element of uncertainty, to be considered worth conducting and this means that the results cannot be predicted or assumed until the analysis is complete. We found a lack of appreciation for equipoise which resulted in an assumption that the intervention would lead to overall benefit [39, 40]. This resulted in what we interpreted to be an overestimation of benefit and an underestimation of risk. Alongside, there was a limited understanding of the concept of randomisation, that there is an equal probability of receiving one of two or more interventions, including a placebo or the best available routine care [42]. As a result, participants were found to be making decisions based on the assumption that they would be receiving the intervention rather than the control arm [33, 41]. In some situations, participants thought that they were being invited to choose one of several different treatment options [35]. In others, where there was an understanding of randomisation but they were randomised to the control arm, some felt ‘let down’ [41] whilst others thought this meant that they had not been ‘chosen for the trial’ [42]. We did not identify any discussions about the blinding process and only two trials used placebos which were not discussed in the qualitative papers [37, 43]. When considering all of the above, in the context of a life-threatening illness, there is a possibility for individuals to make decisions based on an underestimation of risk and an overestimation of benefit, which is centred on an expectation that the intervention will work and that they will receive it.

Another way the severity of the situation was interpreted to exacerbate the experience of those involved in research studies was a difficulty in differentiating research from routine care [36]. As these individuals were being managed in a hospital setting, they explained that in the emergency situation they are in an unusual environment and meet a lot of new people [44]. It was therefore not always possible to disentangle what was being provided as part of routine care and what was part of research, as well as who was providing it. This lack of differentiation made it hard to then pull apart the research from routine care when providing testimonies about being in the research study.

### Recommendations for improvement

The studies included were primarily focused on decision-making and the experience of being in a clinical study rather than specifically aiming to identify areas for improvement. It was however possible to extract data which focused on this, and we identified two core areas for development. The first relates to the formal aspects of the consent process, particularly with regard to how and when this takes place and using which documents. It was felt that consent took place at the most intense time when all of the impairments caused by the life-threatening illness were heightened and, as discussed, the ability to fully understand, retain, and communicate information was at its lowest [42]. It was regularly cited that the information conveyed during this process was too extensive and detailed, particularly in terms of what was written on consent forms, and that a simplified or abbreviated form of consent would be preferred [35, 38, 41]. Another reason for this was that the consent process was seen to delay the treatment which was in many cases potentially lifesaving [40]. Several studies concluded that a shorter summary of the study should be provided whereby more time could be spent conveying the most important information [38]. Consent was viewed as a single, one-off event and some participants felt that it would have been beneficial to have the opportunity to review that decision and discuss further with members of the research team as additional questions or concerns often arose in the following days. In studies where this was offered by the research team, it was appreciated [37]. Some individuals expressed feeling deserted by research teams who recruited and treated them on day 1 at the height of their illness and from their recollection were never seen or heard from again [50]. In these contexts, the consent process was felt to be more of a legal procedure designed to protect the researchers rather than the participants [29].

The second area for development was regard to the communication skills of researchers. Effective, professional, and dignified communication was felt to be critical [41]. This follows on from the above regarding the consent process which could have been improved by researchers taking time to explain the key information in a clear way and then being available for ongoing discussions around the study [45]. In addition, our interpretation of the data was that at times the research teams tended to indirectly convey an assumption that the intervention would be of benefit to the individual which would further exacerbate the lack of understanding of both equipoise and randomisation. This occurred both during the consenting process but also later on when considering the individual participant outcome outside of the context of the final results: for example, attributing an improvement in symptoms or a better outcome to the intervention [36].

## Discussion

Within this review, we have been able to critically interpret and synthesise data from a broad range of settings related to the experience of being enrolled in clinical research when suffering from a life-threatening illness. We have shown that the severity of the illness has a significant impact on all aspects of this experience, particularly the decision-making process. Individuals making decisions are either themselves directly experiencing or witness to an unfolding emergency which comes with a myriad of physical and psychological symptoms. When combined with limited previous knowledge or experience of clinical research, this can result in difficulty comprehending core concepts and the pertinent details of a specific study which can in turn lead to an underestimation of risk, an overestimation of benefit, and an expectation of being allocated to the intervention arm. This is also exacerbated by a difficulty in differentiating clinical research and routine care.

A core theme that emerged related to trust in research teams, institutions, and governance. When faced with a life-threatening emergency, and with limited previous knowledge or experience of clinical research, we found that a great deal of trust was placed in clinical researchers and this was sometimes an acceptable alternative to understanding. These findings emphasise the huge responsibility that researchers have and the need to provide unbiased information that does not unduly influence or pressure individuals into participation. Research concepts are complicated, and the nuances of a study can be particularly so; however, we found a clear preference among decision-makers to be continuously engaged by researchers throughout the duration of a study and to regularly provide information in manageable, bite-size portions. This could be in the form of an abbreviated summary of a study when it is first introduced, outlining the pertinent information, and then providing aftercare: regular, ongoing interaction between participants and researchers throughout the trial process where the information is relayed again and participants are provided with continuous opportunities to seek clarification, re-confirm consent, and opt to withdraw from the study.

The conventional, one size fits all approach of providing all the information in a single, written form upon enrolment was clearly inadequate. The use of a variety of tools, including summaries and visual information, can help to increase understanding. A systematic review of audio-visual consent practices in high-income countries was limited by poor reporting of data but identified trends with regard to improvements in knowledge obtained and satisfaction with the process [51]. A core component of any further research into informed consent is the need for well-defined outcomes for evaluating interventions, for example, those which have been proposed by researchers as part of the ELICIT study [52].

Further research around the best way to optimise both understanding of and satisfaction with the consent process is needed. A number of randomised controlled trials of different approaches to informed consent have been conducted or are underway [53]; however, these have not been in the context of individuals hospitalised in an emergency and this critical interpretive synthesis has clearly highlighted the nuances of this situation. One area where research is increasing however is with individuals who lack the capacity to consent, most often due to cognitive impairment or intellectual disabilities [54]. There also remains a significant gap in the literature whereby most of the research around this subject and the interventions developed as a result have been based in high-income settings. This was exemplified in this critical interpretive synthesis where only three of the included studies were conducted in lower and middle-income countries. Finally, although we reviewed data from decision-makers for paediatric patients, there were no data from those who took part and this may be possible where participants are older and able to communicate or potentially further down the line as they become more mature.

There were some limitations to this review. We adapted the methodology first described by Dixon-Woods which has itself been subject to variation by other researchers and therefore our methods may not be entirely comparable with other critical interpretive syntheses; however, this adaptation was justified throughout the process and any changes were made to fit within our research question and the evolving analysis. Second, life-threatening illnesses and experience vary significantly. We tried to group them together because we felt individuals were facing a similar sociological context but some of the heterogeneity within this group may have been lost. In addition, we did not compare studies of life-threatening illnesses with those which were not life-threatening. Third, as previously discussed, there was a lack of data from lower and middle-income countries, so our interpretation may be less generalisable for these settings; however, we did attempt to emphasise the differences within our analysis. Fourth, there was a small number of observational studies included in the synthesis, and where observational and interventional studies were combined, the findings were not disaggregated by study design, which limits the generalisability of our conclusions about observational research. Finally, one of our key findings was that individuals struggled to differentiate research from routine care when providing testimonies about being in the research study. It is therefore possible that some of the observations and interpretations provided by informants were actually related to routine care rather than research.

## Conclusion

Within this critical interpretive synthesis, we have developed a synthetic construct which aims to outline the experience of enrolling into a clinical research study whilst suffering from a life-threatening illness. We found most individuals had no previous knowledge or experience with clinical research. The decision-making process was hugely impacted by the physical and psychological impact of the life-threatening illness. It was difficult to differentiate clinical research and routine care, and understanding of core concepts around research was limited. This led to an underestimation of risk, an overestimation of benefit, and an expectation of being allocated to the intervention arm. We found that the decision-making process was heavily influenced by trust in the research team. Finally, we provide some suggestions for further research and implementation work around informed consent for individuals suffering from a life-threatening illness.

## Supplementary Information

**Additional file 1: Table S1.** Search strategy.

**Additional file 2: Table S2.** Data extraction form.

**Additional file 3: Table S3.** Summary of characteristics of the included articles.

## Data Availability

The data extraction forms for each included study are available from the corresponding author on reasonable request.
