# Supplementary material for: Clinical research for life-threatening illnesses requiring emergency hospitalisation: a critical interpretive synthesis of qualitative data related to the experience of participants and their caregivers
Source: Trials. 2023 Feb 28;24:149. doi: 10.1186/s13063-023-07183-6 (PMC9972707; doi:10.1186/s13063-023-07183-6)
Supplement: Supplementary file 1 — Additional file 1: Table S1. Search strategy. [file 13063_2023_7183_MOESM1_ESM.docx]

**Table S1: Search strategy**

| LIFE THREATENING | |
| --- | --- |
| 1 | Life threatening |
| 2 | Critical care |
| 3 | Emergency |
| 4 | Exp ‘emergency care’, ‘emergency medicine’, ‘emergency patient’, ‘emergency surgery’, ‘emergency treatment’, ‘emergency ward’, ‘pediatric emergency medicine’, ‘emergency’, ‘evidence based emergency medicine’, ‘hospital emergency service’, ‘obstetric emergency’ |
| 5 | Death |
| 6 | Exp ‘brain death’, ‘fetus death’ ‘maternal death’, ‘newborn death’, ‘sudden cardiac death’, ‘sudden death’, ‘sudden infant death syndrome’, ‘parental death’ |
| 7 | Meningitis |
| 8 | Exp ‘bacterial meningitis’, ‘cryptococcal meningitis’, ‘meningitis’, ‘pneumococcal meningitis’, ‘tuberculous meningitis’ |
| 9 | Stroke |
| 10 | Exp ‘cerebrovascular accident’ |
| 11 | Myocardial infarction |
| 12 | Exp ‘heart infarction’ |
| 13 | Pneumonia |
| 14 | Exp ‘pneumonia’ |
| 15 | Combine 1-14 OR |
| CLINICAL STUDIES | |
| 16 | trial |
| 17 | exp ‘Clinical Trial’, ‘Clinical Trial, Phase I’, ‘Clinical Trial, Phase II’, ‘Clinical Trial, Phase III’, ‘Clinical Trial, Phase IV’, ‘Randomized Controlled Trial’ |
| 18 | randomi#ed trial |
| 19 | prospective |
| 20 | exp ‘Prospective Studies’ |
| 21 | Cohort |
| 22 | Exp ‘cohort analysis’, ‘controlled study’ |
| 23 | Case control |
| 24 | Exp ‘case control study’ |
| 25 | Observational |
| 26 | Exp ‘observational study’ |
| 27 | Combine 16-26 OR |
| QUALITATIVE DATA | |
| 28 | Qualitative |
| 29 | Exp ‘qualitative analysis’, ‘qualitative research’ |
| 30 | Interview |
| 31 | Exp ‘interview’, ‘semi structured interview’, ‘structured interview’ |
| 32 | Focus group |
| 33 | Exp ‘focus group’ |
| 34 | Ethnograph* |
| 35 | Exp ‘ethnography’ |
| 36 | Observation |
| 37 | Exp ‘non-participant observation’, ‘observation’, ‘participant observation’ |
| 38 | Combine 28-37 OR |
| EXPERIENCE | |
| 39 | Experience |
| 40 | Exp ‘experience’, ‘near-death experience’ |
| 41 | Perspective |
| 42 | Feedback |
| 43 | Opinion |
| 44 | Belief |
| 45 | Combine 39-44 OR |
| Combine | |
| 46 | Combine 15 AND 27 AND 38 AND 45 |
