# Supplementary material for: Clinical research for life-threatening illnesses requiring emergency hospitalisation: a critical interpretive synthesis of qualitative data related to the experience of participants and their caregivers
Source: Trials. 2023 Feb 28;24:149. doi: 10.1186/s13063-023-07183-6 (PMC9972707; doi:10.1186/s13063-023-07183-6)
Supplement: Supplementary file 2 — Additional file 2: Table S2. Data extraction form. [file 13063_2023_7183_MOESM2_ESM.docx]

**Table S2: Data extraction form**

| **BIBLIOGRAPHIC INFORMATION** | |
| --- | --- |
| Title |  |
| Authors |  |
| Research institutions listed |  |
| Article type (e.g. original research / report) |  |
| Journal |  |
| Publication Year |  |
| Country of setting |  |
| **AIMS, METHODS AND PARTICIPANTS** | |
| Name of clinical study in which this was embedded |  |
| Type of study (trial, cohort etc) |  |
| Disease under investigation |  |
| Population under investigation |  |
| Intervention(s) (if applicable) |  |
| Qualitative study aims |  |
| Specific objectives / research questions |  |
| Theoretical and epistemological perspective underpinning the research |  |
| Inclusion criteria |  |
| Exclusion criteria |  |
| Sampling |  |
| Data collection method(s) |  |
| Data collection location (e.g. hospital, clinic, telephone) |  |
| Time period data collected over |  |
| Categories of participants |  |
| Number of participants |  |
| Timeframe in relation to the clinical study (collected in-situ, after the trial etc) |  |
| Data handling methods (transcription, translation, verification etc) |  |
| Data analysis methods |  |
| **FINDINGS** | |
| Theme 1 |  |
| Summary of theme 1 |  |
| Primary data to support theme 1 |  |
| Theme 2 |  |
| Summary of theme 2 |  |
| Primary data to support theme 2 |  |
| Theme 3 |  |
| Summary of theme 3 |  |
| Primary data to support theme 3 |  |
| Theme 4 |  |
| Summary of theme 4 |  |
| Primary data to support theme 4 |  |
| Theoretical Development |  |
| Figures/Thematic Networks |  |
| Conclusions |  |
| **QUALITY ASSESSMENT** | |
| Are the aims and objectives of the research clearly stated? |  |
| Is the research design clearly specified and appropriate for the aims and objectives of the research? |  |
| Do the researchers provide a clear account of the process by which their findings we reproduced? |  |
| Do the researchers display enough data to support their interpretations and conclusions? |  |
| Is the method of analysis appropriate and adequately explicated? |  |
| Notes on generalisability |  |
| Notes on reflexivity and the role of the researcher |  |
| Were any other potentially useful references listed in the bibliography? |  |
| General thoughts of the reviewers |  |
| **Reviewer One** |  |
| **Date** |  |
| **Reviewer Two** |  |
| **Date** |  |
