# Supplementary material for: Clinical research for life-threatening illnesses requiring emergency hospitalisation: a critical interpretive synthesis of qualitative data related to the experience of participants and their caregivers
Source: Trials. 2023 Feb 28;24:149. doi: 10.1186/s13063-023-07183-6 (PMC9972707; doi:10.1186/s13063-023-07183-6)
Supplement: Supplementary file 3 — Additional file 3: Table S3. Summary of characteristics of the included articles. [file 13063_2023_7183_MOESM3_ESM.docx]

**Table S3:** **Summary of characteristics of the included articles**. GWAS denotes Genome Wide Association Study; ICU denotes Intensive Care Unit; RCT denotes Randomised Controlled Trial and SDM denotes Surrogate Decision Maker.

| **Study** | **Location** | **Embedded within** | **Clinical study population** | **Disease/s** | **Qualitative population** |
| --- | --- | --- | --- | --- | --- |
| **Agard et al.** (2001) | Sweden | RCT | Adults | Myocardial infarction | Participants |
| **Burns et al.** (2015) | Canada | RCT | Adults | Not specified but in ICU | SDMs |
| **Burns et al.** (2017) | Canada | Various | Adults | Not specified but in ICU | SDMs and decliners |
| **Chatio et al.** (2016) | Ghana | RCT | Children | Acute malaria | SDMs |
| **Dickert et al.** (2015) | USA | RCT | Adults | Myocardial infarction | Participants |
| **Dotolo et al.** (2017) | USA | RCT | Adults | Not specified but in ICU | Participants, SDMs and decliners |
| **Gammelgaard et al.** (2004) | Denmark | RCT | Adults | Myocardial infarction | Participants and decliners |
| **Houghton et al.** (2018) | UK | RCT | Adults | Post-partum haemorrhage | Participants |
| **Lawton et al.** (2016) | UK | RCT | Adults | Retained Placenta | Participants, research staff |
| **Lawton et al.** (2017) | UK | RCT | Adults | Retained placenta | Participants |
| **Mangset et al.** (2008) | Norway | RCT | Adults | Stroke | Participants |
| **Molyneux et al.** (2013) | Kenya and Uganda | RCT | Children | Severe febrile illness and shock | SDMs |
| **Scicluna et al.** (2019) | USA | RCT | Adults | Myocardial infarction or stroke | Participants |
| **Snowdon et al.** (1997) | UK | RCT | Neonates | Extracorporeal membrane oxygenation | SDMs |
| **Snowdon et al.** (2006) | UK | RCT | Neonates | Neonatal related conditions | SDMs |
| **Snowdon et al.** (2014) | UK | RCT | Neonates and Children | Multiple but all life-threatening | Bereaved SDMs, clinicians, trial team members |
| **Thomas et al.** (2013) | Canada | Various | Children | Not specified but in ICU | SDMs |
| **Tindana et al.** (2012) | Ghana | GWAS | Children | Malaria | SDM and researchers |
| **Tutton et al.** (2018) | UK | RCT | Adults | Open fracture lower limb | Participants and one decliner |
| **Van den Berg et al.** (2017) | UK | RCT | Adults | Acute coronary syndrome | Participants |
| **Veron et al.** (2018) | Switzerland | RCT | Adults | Chronic obstructive pulmonary disease | Participants |
| **Ward et al.** (2009) | USA | Not stated | Neonates | Not specified but in ICU | SDMs |
